# TraMiner: Vision-Based Analysis of Locomotion Traces for Cognitive Assessment in Smart-Homes

**DOI:** 10.1007/s12559-020-09816-3

**Published:** 2021-02-02

**Authors:** Samaneh Zolfaghari, Elham Khodabandehloo, Daniele Riboni

**Affiliations:** 1 Department of Mathematics and Computer Science, University of Cagliari, Cagliari, Italy; 2 Department of Geo-spatial Information Systems, K. N. Toosi University of Technology, Tehran, Iran

**Keywords:** Pervasive healthcare, Artificial intelligence, Cognitive decline, Deep learning

## Abstract

The rapid increase in the senior population is posing serious challenges to national healthcare systems. Hence, innovative tools are needed to early detect health issues, including cognitive decline. Several clinical studies show that it is possible to identify cognitive impairment based on the locomotion patterns of the elderly. In this work, we investigate the use of sensor data and deep learning to recognize those patterns in instrumented smart-homes. In order to get rid of the noise introduced by indoor constraints and activity execution, we introduce novel visual feature extraction methods for locomotion data. Our solution relies on locomotion trace segmentation, image-based extraction of salient features from locomotion segments, and vision-based deep learning. We carried out extensive experiments with a large dataset acquired in a smart-home test bed from 153 seniors, including people with cognitive diseases. Results show that our system can accurately recognize the cognitive status of the senior, reaching a macro-$F_1$ score of 0.873 for the three categories that we target: cognitive health, mild cognitive impairment, and dementia. Moreover, an experimental comparison shows that our system outperforms state-of-the-art methods.

## Introduction

Nowadays, different societal factors are determining a significant reduction in fertility and an increase in life expectancy. As a consequence, we are observing a rapid and constant growth of the average population age. This phenomenon determines increasing pressure on healthcare systems. Indeed, more and more seniors affected by noncommunicable diseases (NCDs) need to be hospitalized for long periods, and this trend is projected to continue for the foreseeable future [1]. Unfortunately, the recent COVID-19 pandemic has dramatically exposed the fragility of national healthcare systems when dealing with increasing numbers of patients [2], and the need for innovative technological solutions to cope with global health crises has emerged. Among the NCDs that affect the elderly population, one of the most prevalent is dementia. For instance, the prevalence of dementia is estimated to $$14\%$$ in the U.S. population aged 70 or older, with a total cost estimated from $157 billion to $215 billion, and this cost is projected to triple by 2050 [3]. Of course, in addition to monetary costs, dementia and other cognitive issues disrupt the quality of life of patients and of those caring for them. Hence, in order to prolong independent and healthy aging, it is urgent to devise novel tools to continuously monitor the mental wellness of seniors and to early detect abnormal cognitive decline in an unobtrusive and privacy-conscious manner.

In this paper, we contribute to this grand challenge by proposing *TraMiner*, a novel system based on Trajectory Mining for continuous cognitive assessment in smart-homes. Our system relies on clinical indicators that characterize cognitive decline in terms of abnormal locomotion patterns of the elderly. Different solutions have been recently proposed to recognize those patterns outdoors, including [4–6]. However, most seniors, and especially those experiencing cognitive issues, normally spend a large part of their time at home. Hence, indoor locomotion traces provide invaluable information for assessing their cognitive health status. Unfortunately, recognizing those patterns at home is challenging, since the inhabitant movements are constrained by the home environment, and they are affected by the execution of daily living activities. The problem of indoor abnormal pattern detection for cognitive assessment has been investigated in a few previous works. Up to date, most of those works rely on manual feature engineering to define statistical measures characterizing locomotion traces [7–9]. However, locomotion traces encode involved spatio-temporal information, which can be only partially captured through a fixed set of numerical features. For this reason, we take a different approach, representing locomotion traces through images and relying on Deep Learning (DL) for the classification of those images according to the cognitive health status of the inhabitant. Our method relies on specific techniques for locomotion data cleaning and segmentation. Each locomotion segment is transformed into two images that capture different aspects related to the clinical indicators of locomotion anomalies. Each couple of images is fed to a DL classifier in charge of predicting the anomaly level of the corresponding locomotion segment. Since it would be unrealistic to provide a hypothesis of diagnosis based on the observation of a single trajectory, our system includes a long-term analysis module. The latter is in charge of processing the history of DL predictions for computing a hypothesis of diagnosis, which is provided to clinicians for supporting the neuropsychological assessment.

We have developed a prototype of TraMiner system, and carried out extensive experiments with a real-world dataset gathered in a smart-home test bed with 153 seniors, including cognitively healthy subjects, people with Mild Cognitive Impairment (MCI) [10], and people with dementia (PwD). Results proved the accuracy of TraMiner in predicting the cognitive health status of the inhabitants. Indeed, based on the observation of the locomotion traces acquired during one day per patient, our system achieved a macro-$F_1$ score of 0.873. Moreover, an experimental comparison showed the superiority of our approach with respect to state-of-the-art techniques. In order to enable the full reproducibility of our experiments, we have released the code of all our system components, and the used dataset is available on the Web.

A preliminary investigation of this work was presented in [11]. In this paper, we extend our preliminary investigation with: (i) support for recognition of MCI, (ii) a novel locomotion trace segmentation algorithm, (iii) a new visual feature extraction technique, (iv) a novel two-input DL architecture, (v) an additional algorithm for long-term assessment of the cognitive health status, (vi) experiments with a larger dataset, (vii) new experiments to evaluate all the components of our system, (viii) an experimental comparison with state-of-the-art techniques, and (ix) a dashboard accessible on the Web to inspect the visual features and the system predictions.

The rest of the paper is structured as follows. Section 2 introduces preliminary notions and discusses related work. Section 3 illustrates our overall system. Section 4 describes the techniques for trajectory segmentation and visual feature extraction. Section 5 explains our methods for DL-based trajectory classification and long-term analysis. Section 6 reports experimental results. Finally, Section 7 concludes the paper and outlines future research directions.

## Preliminaries and Related Work

In this section, at first, we illustrate different clinical indicators to recognize cognitive decline based on locomotion data, which are the theoretical basis of our work. Then, we review existing techniques based on Internet of Things (IoT) technologies to automatically detect abnormal locomotion patterns that may indicate cognitive decline.

### Clinical Indicators of Abnormal Locomotion Patterns

Several studies have attempted to characterize the typical locomotion patterns of people experiencing cognitive decline. Some models relied on the notion of *wandering*, defined by Algase et al. [12] as a *”syndrome of dementia-related locomotion behavior having a frequent, repetitive, temporally disordered, and/or spatially disoriented nature that is manifested in lapping, random, and/or pacing patterns”*. Other researchers characterized cognitive decline based on the observation of subtle gait anomalies. In the following, we present the models adopted in this work.

#### Martino-Saltzman Model

**Fig. 1:**
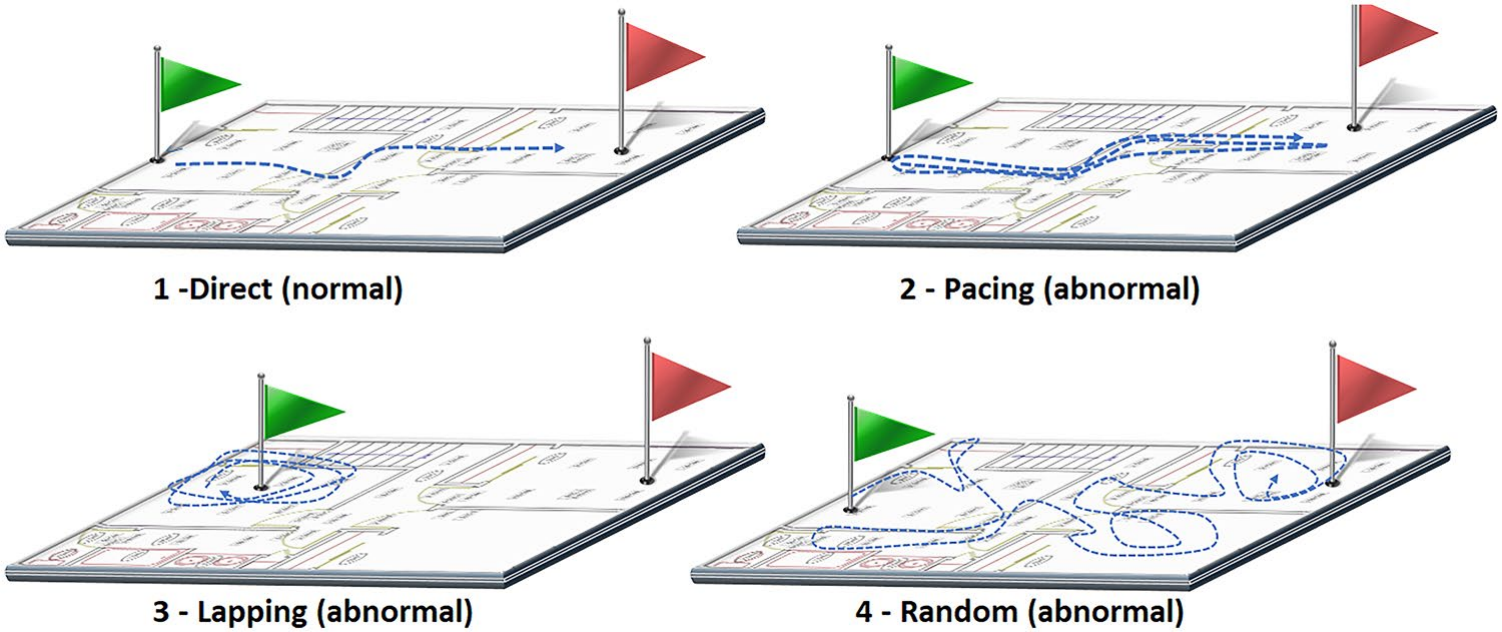
Travel patterns of people according to the Martino-Saltzman model of wandering behavior [13]

One of the most well-known and widely accepted models for categorizing the patterns of wandering behaviors in dementia was proposed by Martino-Saltzman [13]. This model categorizes the trajectories into one of four distinctive patterns of movement: direct, random, lapping, or pacing, which are shown in Fig. 1:

- **Direct**: a simple or uncomplicated trajectory from one location to another one.
- **Pacing**: at least three consecutive back-and-forth movements between two locations along very similar paths.
- **Lapping**: at least two circular movements between at least three distinct locations.
- **Random**: a continuous and aimless movement across numerous locations with multiple directional changes, that generally passes through more than four locations.

Based on the Martino-Saltzman model, *random*, *pacing*, and *lapping* patterns are indicators of cognitive issues. As explained in Introduction, several random, pacing, and lapping patterns can be observed in the home, which are actually due to the normal execution of daily living activities by cognitively healthy inhabitants. Hence, in this work, we do not aim at explicitly recognizing those patterns, but we rely on a supervised learning approach for recognizing abnormal locomotion patterns.

#### Low-Level Motion Indicators

In addition to clinical indicators based on wandering behavior, different low-level indexes were introduced in other works as indicative of cognitive decline. Those indicators have been used to automatically assess the cognitive status in IoT systems [14]:

- **Jerk** [15] is the rate at which a person’s acceleration changes with respect to time.
- **Sharp angles** [6] are defined as vector angles in a trajectory being equal to or more than 90 degrees.
- **Straightness** [16] is defined as the ratio of the distance between two consecutive trajectory segments and the distance between the start and end point of these segments.
- **Turning angle** [9] is defined as the sum of the absolute angles between any two subsequent lines in a trajectory.
- **Path efficiency** [9] is defined as the ratio between the distance from the start to the end of a trajectory and the trajectory length.

### IoT Techniques for Detecting Locomotion Anomalies

In the last few years, different methods have been proposed to recognize cognitive issues based on artificial intelligence (AI) [17], and several AI-based solutions have been proposed to support active and healthy aging [18–20].

Riboni et al. relied on activity recognition algorithms to detect overt errors in the execution of activities of daily living, which indicate cognitive decline [21]. Other works, including those of Seelye et al. [22] and Dawadi et al. [23], rely on sensor activations and activity patterns to distinguish PwD subjects from cognitively healthy ones. However, activity recognition systems determine privacy issue, especially when deployed in homes. Moreover, those systems require a dense sensing infrastructure, while in this work we rely solely on an indoor positioning system.

Indeed, a promising direction consists in tracking the movements of the elderly through unobtrusive positioning technologies, and analyzing location traces according to clinical indicators as the ones mentioned in Section 2.1. Methods based on low-level motion indicators usually rely on body-worn sensors. Mc Ardle et al. used accelerometers worn by the senior to detect symptoms of cognitive impairment [24]. In that work, the authors extracted spatiotemporal features including gait variability, rhythm, asymmetry, and postural aspects of gait. An accelerometer-based method to detect challenging behaviors typical of PwD was proposed by Goerss et al. [25]. That method relies on the extraction of actimetric movement features from raw sensor data. Experiments carried out with 17 seniors in a retirement home showed that the long-term observation of those features provided relevant information to identify the challenging patterns of PwD. The correlation between low-level motion behaviors and dementia was assessed in different studies, including the one carried out by Kirste et al. relying on ankle-mounted three-axis accelerometers [26]. However, body-worn systems determine usability issues, especially for the senior population [27]. Hence, in our work we disregard body-worn sensors, and we rely on fixed positioning infrastructures to detect indoor location traces.

Most existing works aimed at recognizing cognitive impairment based on locomotion patterns rely on outdoor movements. Lin et al. [6] analyzed GPS trajectories outdoors for detecting wandering according to the Martino–Saltzman model. A smart GPS tracker was devised by Ng and Kong for supporting secure and independent outdoor walking of the elderly [5]. That system is based on wandering detection and activity recognition and can adapt to personal locomotion wandering patterns. Schaat et al. investigated the use of GPS and accelerometer data for real time detection of disorientation in PwD [4].

Since many elderly people, and especially those who are experiencing cognitive decline, spend large part of their time at home, indoor locomotion data can be a rich source of information to assess their cognitive status. Unfortunately, indoor wandering recognition is particularly challenging, since movements are constrained by the ambient shape, and are impacted by the execution of activities of daily living. A few previous works have addressed this issue. Vuong et al. applied supervised machine learning methods for automatically classifying indoor trajectories according to the Martino–Saltzman model [28]. Lin et al. proposed a method to identify repetitive locomotion episodes in the home according to well-known models of wandering [29]. Khodabandehloo and Riboni proposed a collaborative mining approach to assess the cognitive status of smart-home inhabitants based on statistical features extracted from their trajectories [7]. Kearns et al. proposed the use of accurate localization technologies deployed in a common indoor living space of a retirement home, and measured the tortuosity of trajectories to recognize wandering episodes [8]. In a subsequent study on the same setup, the authors found out that other features, including speed, path efficiency, and angle-turn, were predictive of dementia [9]. Other studies showed that the relation between in-home walking velocity and activity patterns was significantly correlated to the cognitive status of the inhabitant [30].

However, feature engineering from trajectory data is not a trivial task. Indeed, the abundance of spatiotemporal information encoded by movement traces, and the presence of noise introduced by positioning technologies, make it challenging to summarize trajectory data through numerical features. In order to overcome the feature engineering challenge, a recent research direction consists in representing complex information through images, and using Deep Neural Networks (DNN) for images classification to solve the AI task. This approach is used in different domains, including financial forecasting [31], activity recognition [32], and predictive maintenance [33], just to name a few. In this paper, we pursue this approach. The rationale of our choice is that trajectory images may effectively capture discriminative features without the need of sophisticated feature engineering efforts. This approach was applied to trajectory data in few previous studies. In a recent work, Wang et al.  [34] proposed an action recognition method based on the representation of 3D skeleton sequences as 2D images. Sequences of movements are represented using different color distributions. They used DL, in particular, convolutional neural network (CNN), to detect human actions. Kieu et al. used trajectories in the form of 3D images containing geographical features, together with driving behavior features, to predict the identity of drivers [35]. They applied unsupervised auto encoder neural networks to avoid over-fitting, and supervised neural networks to identify drivers. Endo et al. used DNN as an automatic feature extraction method, along with handcrafted features, for estimating users’ transportation modes from images depicting their movement trajectories [36]. Transportation modes were then recognized using supervised learning algorithms.

In this paper, we investigate the application of image-based trajectory classification to the domain of cognitive assessment. To the best of our knowledge, the only previous work that adopted a similar approach to the same domain was proposed by Gochoo et al. in [37]. In that work, the authors represent trajectories in a two-dimensional grid, in which one dimension represents time, while the other dimension represents mono-dimensional space. They map each three-dimensional spatiotemporal trajectory point into the two-dimensional grid. The corresponding binary image is then classified using deep convolutional neural networks to recognize different patterns of wandering behavior. However, as a consequence of the transformation from two-dimensional to one-dimensional space, the spatial information is partially disrupted, since metric operations and topological relationships are not preserved. Indeed, points that are close in the two-dimensional geographic space are not necessarily close in the one-dimensional space of the grid.

In our work, we pursue a different direction, retaining the spatial information in trajectories images, and enriching them with additional visual features encoding low-level motion indicators of cognitive decline. To the best of our knowledge, apart from the preliminary investigation that we carried out in [11], this is the first work that investigates this method for cognitive assessment.

## TraMiner System Overview

In this paper, we assume a smart-home infrastructure capable of continuously monitoring the inhabitant’s position at a fine-grained level. For the sake of this work, we consider the case of a person living alone in the home. This is a common situation for elderly people. Moreover, seniors living alone may have particular benefits from remote monitoring and assessment of cognitive functions. In order to support seniors living with other people or pets, our system could be easily extended by adopting an identity-aware positioning system, or by applying a *data association* algorithm in charge of associating each location reading to the individual that triggered the corresponding sensor [38]. Our system may extract position information not only from localization infrastructures, but also based on the inhabitant’s interaction with sensorized objects and appliances with a fixed position in the home.

**Fig. 2:**
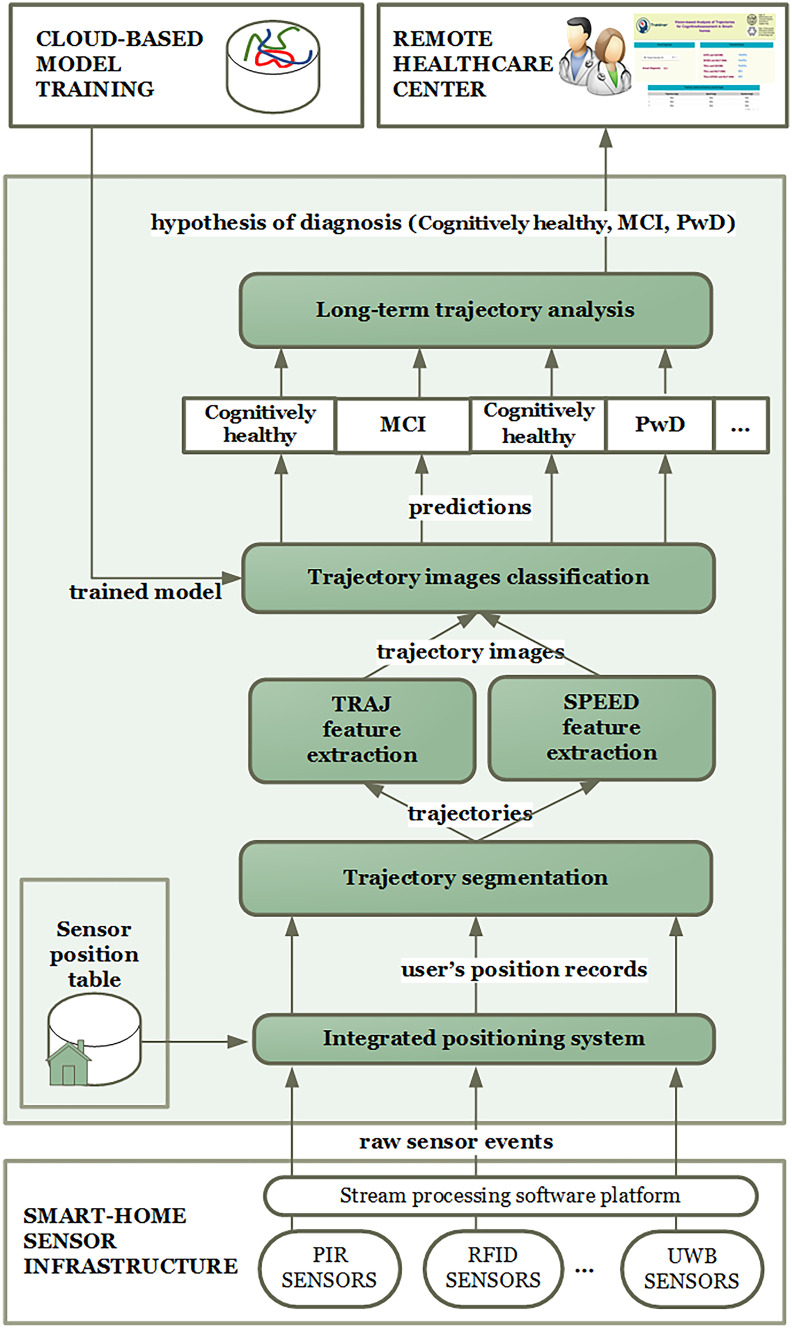
Overview of the TraMiner system

Figure 2 illustrates the TraMiner architecture. Since the focus of this paper in on processing locomotion data, the core methods of our contribution are largely independent from the available sensor infrastructure. We assume that a smart-home sensor infrastructure is in charge of continuously gathering data about the inhabitant’s position, e.g., through passive infrared (PIR) sensors. The smart-home system communicates raw sensor data to a stream processing software platform (e.g., Apache Kafka) for integration and temporal synchronization.

Each time a sensor fires, the platform sends a **raw sensor event** $rse = \langle t, s\_id, v \rangle$ to the TraMiner system, where *t* is the timestamp of firing, $s\_id$ is the sensor’s unique identifier, and *v* is the generated value. For instance, the raw sensor event:

$$\begin{aligned} rse = \langle \textit{sens5371, 2020-08-30 16:46:18.323, open} \rangle , \end{aligned}$$

states that the sensor identified as ’sens5371’ (for instance, a sensor attached to the fridge door) fired a value ‘open’ at timestamp ‘2020-08-30 16:46:18.323’. Since we assume that the home is inhabited by a single individual, we are not interested in associating the sensor record to the person that triggered it.

The integrated positioning system is in charge of deriving spatiotemporal information from raw sensor events. To this aim, it relies on a **sensor position table** storing the relative position of each sensor in the home. A record of sensor position in that table is a triple: $\langle s\_id, x, y \rangle$, where (*x*, *y*) are the relative coordinates of the sensor identified by ’$s\_id$’ in the home. For the sake of simplicity, we consider only static sensors in the home; hence, we assume that the position of sensors does not change with time. Each time the integrated positioning system receives a raw sensor event, it joins the corresponding record with the sensor position table to obtain the (*x*, *y*) coordinates, producing a **user’s position record** $r = \langle p, t \rangle$, where $p=(x,y)$ are the relative coordinates of the sensor that fired at time *t*.

The trajectory segmentation module is in charge of reducing noise in the data and partitioning the temporal stream of position records into **trajectories**. A trajectory is a temporally contiguous sequence of positions which corresponds to a locomotion episode. Each trajectory is passed to the modules for traj and speed feature extraction. Those modules represent the trajectory as an image each. The two images represent the walked trajectory in a two-dimensional space and highlight different visual features, such as speed and intersection points.

The trajectory images classification module is in charge of classifying each trajectory as either walked by a cognitively healthy person, MCI individual, or PwD. To this aim, it uses a DL classifier processing the two images corresponding to each trajectory. The classifier is trained on the cloud using a set of anonymous trajectories labeled according to the cognitive health status of the individual. According to the history of predictions, the long-term trajectory analysis module computes a hypothesis of diagnosis for the individual. The hypothesis may be *cognitively healthy*, *MCI*, or *PwD*. Finally, the hypothesis of diagnosis is communicated to the remote healthcare center, to support the clinicians in the evaluation of the patient.

## Trajectory Segmentation and Visual Feature Extraction

This section reports the details of modules in charge of trajectory segmentation and visual feature extraction.

### Position Data Cleaning and Trajectory Segmentation

As explained before, the integrated positioning system continuously provides TraMiner with user’s position records. These records instantiate the **position history** $H = \langle r_1, r_2, \ldots , r_n \rangle$, which is the temporal sequence of user’s positions records. Inevitably, the position history contains inaccuracies because, in real-word conditions, sensor data are affected by a relevant level of noise. In this regard, noise reduction is an inevitable step in preparing the data. For addressing this problem, we manually analysed the spatiotemporal information of the dataset used in our experiments by plotting movement traces over the home layout. We observed several unfeasible deviations from the expected trajectories, which were due to wrong position detection by the positioning system. Hence, the trajectory segmentation module performs the following preliminary steps for noise reduction:

- We set a threshold $T_v$ for the maximum possible velocity *v* of a person moving in the home. If the speed between any two consecutive position records $\langle r_i, r_{i+1} \rangle \in H$ is higher than $T_v$, the record $r_{i+1}$ is considered a noisy reading; hence, it is deleted from *H*. For the sake of this work, we set $T_v$ to $15 \, m/s$.
- By considering the arrangement of sensors in the test-bed of our experiments, the maximum distance between any two adjacent sensors is below $3 \, m$. By carefully analyzing the persons trajectories, we observed that some paths spatially deviated from the expected trajectory due to abrupt movements between non-contiguous sensors. In this regard, if the distance between the positions of two consecutive records $\langle r_i, r_{i+1} \rangle \in H$ exceeds a threshold $T_d$ (which is set to $5 \, m$ in this work), we remove $r_{i+1}$ from *H*.

After position data cleaning, the trajectory segmentation module is in charge of partitioning the denoised *H* into **trajectories**. In this regard, the position history *H* is partitioned into a set *T* of non-overlapping trajectories:

$$\begin{aligned} T = \{ t_1, t_2, \ldots , t_m \}, \end{aligned}$$

where each trajectory $t \in T$ is a temporal sequence of consecutive position records:

$$\begin{aligned} t = \langle p_j, p_{j+1}, \ldots , p_k \rangle \in H. \end{aligned}$$

A trajectory consists of locomotion and non-locomotion phases. The non-locomotion phases happen during a time interval between any two consecutive sensor activations that do not exceed a given threshold $T_s$. In our experiments, we evaluate different values of $T_s$ ranging from 30*s* to 180*s*. The rationale of our segmentation algorithm is that a phase of non-locomotion may happen in a trajectory if no sensor is triggered for less than $T_s$. Otherwise, if no user movement is detected for more than $T_s$, the previous trajectory is completed, and a new one is initialized.

Our segmentation algorithm initializes the first trajectory with the first entry of *H*, and each trajectory $t_i$ is a temporal sequence of consecutive position records from *H* such that the time interval between any two consecutive position records does not exceed $T_s$. The next trajectory is initialized with the last entry of the previous trajectory, and continues until the threshold condition is met. The algorithm continues until the end of *H*.

### Visual Feature Extraction

In order to prepare input data for the DNN, the modules for traj and speed feature extraction are in charge of visually representing the salient features of each trajectory through images. To prepare the images, we use the RGB color model. Hence, each image has three colors: red, green, and blue. For every pixel, the values related to these colors are represented by a byte each. Hence, each pixel is characterized by three integer values ranging from 0 to 255. In our work, the maximum spatial extent of all the trajectories is the same, and corresponds to the home layout.

Choosing the image size is an important aspect for feature extraction. By increasing the resolution of the image, the sparsity of non-zero pixels in the image increases, and may decrease the learning capability of the DNN [36]. Low-resolution images can alleviate this issue; however, important information in the image could be lost, since the values of multiple contiguous pixels could be mixed. After considering different resolutions for the images, for our experimental setup, we chose image length and height of 100 by 130 pixels. By considering the size of our smart-home test bed, each pixel in the image correspond to approximately $0.1 m^2$.

#### TRAJ Feature Extraction

**Fig. 3:**
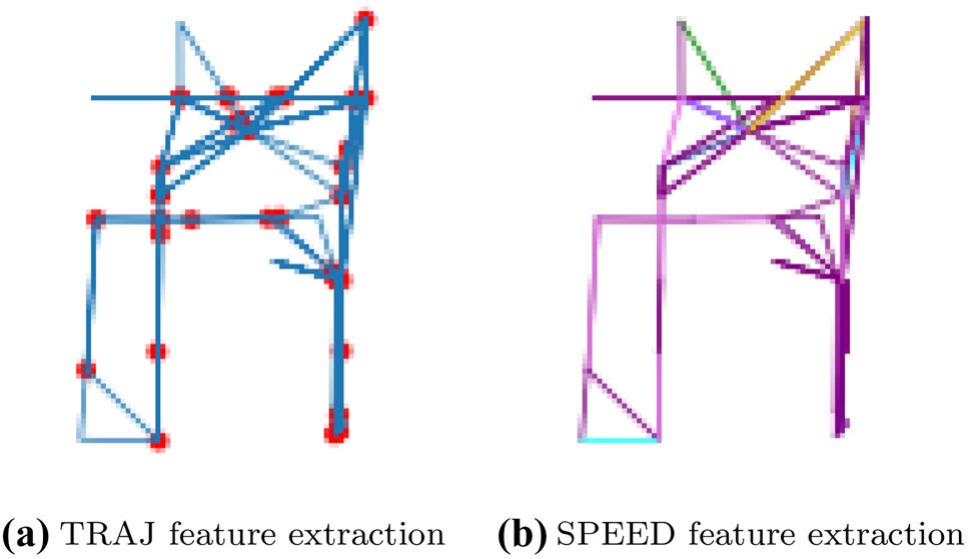
Example of visual feature extraction from a trajectory

Figure 3 shows an example of image obtained using the TRAJ feature extraction method from a person’s trajectory in our smart-home test bed.

The trajectory path is shown through a monochrome line string. The weight of each line is set according to the number of times that the corresponding path has been walked. The line weight is 1 if the path has been walked only once; the weight is 2 if it has been walked twice, and so on. Consequently, lines corresponding to paths walked several times are bolder than those of paths walked sporadically. This feature extraction method allows us to visually capture locomotion anomalies corresponding to the pacing and lapping patterns defined by the Martino–Saltzman model.

For emphasizing the intricacy of the trajectory, which may indicate random walk according to the Martino–Saltzman model, the intersection points between the trajectory lines are shown in the image as a red circle. By considering the image size, the radius of that circle corresponds to 0.25*m*.

Moreover, the trajectory line string naturally encodes low-level motion indicators such as sharp angles, straightness, turning angle, and path efficiency.

#### SPEED Feature Extraction

As explained in Section 2.1.2, patterns of locomotion speed are important indicators of cognitive decline. However, speed information is not captured by the TRAJ feature extraction method.

Hence, TraMiner produces a second image of the trajectory, using the SPEED feature extraction method. Figure 3 shows an image obtained using the SPEED method from a person’s trajectory in our experimental test bed. With this method, the trajectory is partitioned in sections, such as the speed of the inhabitant is steady in each section. The color of each section depends on its speed range. When a section is walked multiple times, the most recent speed of that section is considered for drawing the image. For the sake of this work, we consider seven different speed ranges, with 7 associated colors. In particular, the speed ranges are 0-2, 2-4, 4-6, 6-8, 8-10, 10-12, 12-14, and more than 15 m/s, and the corresponding colors are purple, violet, blue, cyan, green, yellow, orange, and red, respectively. Of course, some of those speed ranges are exaggerated when referred to people moving inside an apartment. The explanation is that the positioning infrastructure used in our experimental test bed has approximately 1 meter resolution, and (being based on passive infrared technology) the detection range of sensors partially overlaps. For this reason, the computed speed is inevitably approximated and subject to errors. However, as confirmed by our experimental results, those approximated speed values are useful to improve the recognition performance of our system. The SPEED feature extraction method allows to capture the jerk low-level motion indicator.

## DNN Trajectory Classification and Long-Term Analysis

In the following of this section, we present the DNN trajectory image classification method and the long-term analysis module.

### Cloud-Based Model Training

The goal of the cloud-based model training module is to train a DNN model to classify every trajectory as either walked by a cognitively healthy subject, by a person with MCI, or by PwD. To this aim, we take a collaborative approach. That module periodically receives a training set of trajectory images from the local instances of the TraMiner system, running in the individual homes. Each image is labeled with the cognitive status of the anonymous inhabitant (i.e., either ’cognitively healthy’, ’MCI’, or ’PwD’). Trajectory images are locally computed on the edge by the different instances of TraMiner, as explained in Section 4. Based on that training set, the trusted cloud-based module is in charge of training a DNN model for trajectory classification. The TraMiner local instances receive the model from the cloud, and use it for classifying the trajectories of the inhabitant. Note that the TraMiner instances do not receive any trajectory image. Indeed, for the sake of privacy, trajectory images are processed only by the trusted cloud module.

**Fig. 4:**
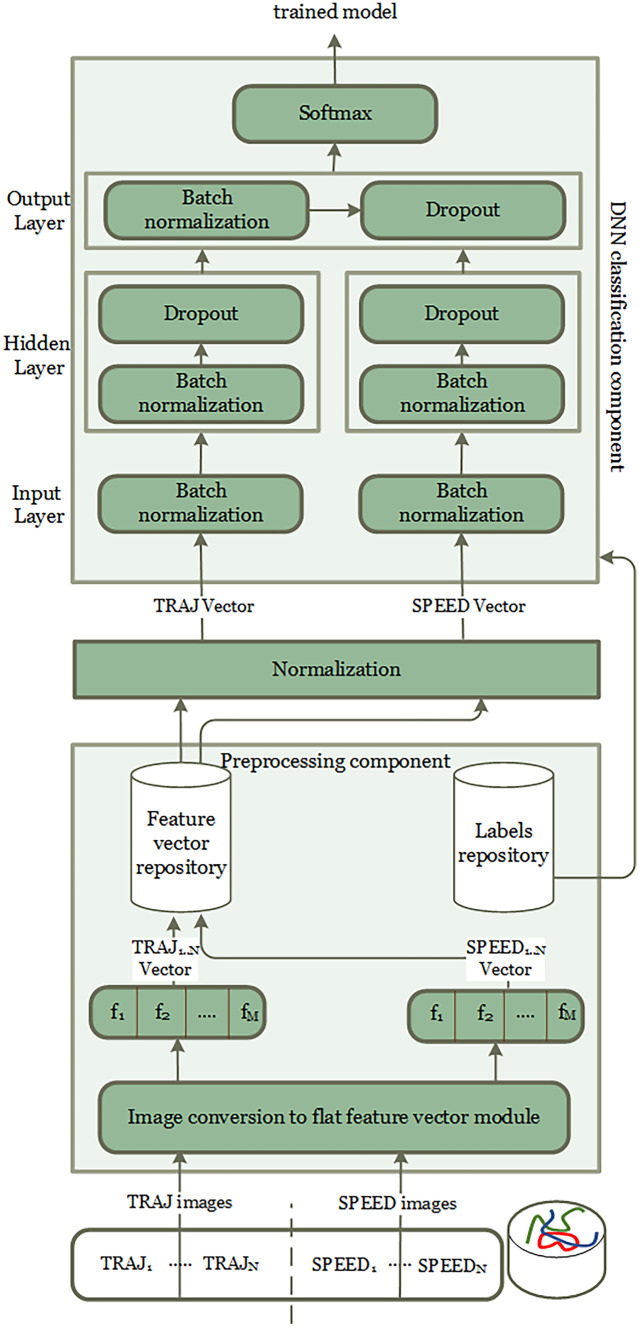
Cloud-based model training MLP DNN architecture

Figure 4 shows the multilayer perceptron (MLP) DNN architecture used by the cloud-based infrastructure. For each trajectory, the MLP DNN takes as input two images. The first one is obtained through the TRAJ feature extraction method as shown in Fig. 3, while the second one is obtained through SPEED feature extraction as shown in Fig. 3. Since we use two images with different features for each trajectory, our model relies on ’mixed data’. Developing systems capable of handling mixed data is still an open area of research, and can be challenging since each input with different feature representation may require separate preprocessing steps. In order to prepare the trajectories images for training, we convert them to flat feature vectors based on their width, height, and color values of each pixel. Hence, since images have 100 by 130 pixels, and each pixel is represented by three color values, each feature vector has size 39,000. So, each feature corresponds to the color value of a single pixel. Then, we apply binarization to the color values of the image to reduce the computational cost of training. Finally, we add the labels to the feature vectors before feeding them to the DNN for training.

The used MLP DNN for each input is composed of two fully connected (dense) layers: one input layer and one hidden layer, with 32 neurons for both. The layers used the Rectified Linear Units function (ReLU) as the activation function. Furthermore, for both inputs each layer is followed by batch normalization to speed up learning and increase the stability of the neural network. In the last layer for each of them, there is also a dropout layer in order to significantly reduce over-fitting. The fraction of drop units has been set to 0.5. Then, we combine the output of both inputs (TRAJ and SPEED) and apply one more fully connected layer with three neurons followed by batch normalization and dropout with same drop units. Since we are facing a three-class classification problem, we have chosen the softmax function as activation function on the final output layer. This function is used to find a probability distribution of the mentioned categories as follows:

$$\begin{aligned} p(k) =\frac{g_s}{\sum _{j=1}^{N_c} g_j}, g_i=max(0,\sum _i f_i.w_{ij} + h_j) \end{aligned}$$

where *p*(*k*) is the probability of a trajectory to belong to the *k*-th class, $g_s$ is a standard exponential function applied to each element of the input vector. This function gives a positive value above zero, which will be very large if the input was large and very small if the input was negative. Furthermore, $N_c$ denotes the number of classes, $f_i$ is a value of *i*th neuron in the last fully connected layer, $w_{ij}$ and $h_j$ are coefficients of the softmax function [37].

Furthermore, we have used a low learning rate, set to 0.00001, with adam optimizer. In this field, this solution achieved major improvements over other regularization methods [39, 40]. As a loss function, we have used category cross-entropy, which is an effective loss function for classification problems which have softmax activation function in the output layer. This loss function is based on the maximum likelihood estimate approach and it is used for single label categorization [41]. In our case, this ensures that a trajectory belongs to exactly one class.

We have used a batch size approach to calculate the model error and to update the model coefficients. We divided the training dataset into small batches of 64 samples which are utilized in one epoch. In order to fine-tune the number of epochs, we have used the *early stopping* procedure. We tested different numbers of epochs, ranging from 20 to 100 epochs. According to this procedure, the training is stopped when the generalization error increases. Therefore, we evaluated the model during training on a holdout validation set after each epoch. When the performance of the model starts to degrade (i.e., the loss begins to increase), the training process is stopped. In the presented configuration, the number of epochs was set to 27. With this approach, we improved the computational efficiency of the learning process, without the need of keeping all training data in the main memory. The approach also provides robust convergence, avoiding local minima [42].

### Long-Term Trajectory Analysis

As shown in Fig. 2, the trained model is communicated to the trajectory images classification module of the individual smart-home systems, which uses it for trajectory image classification as soon as new trajectories are observed in the home. Finally, the classification predictions are processed by the long-term trajectory analysis module, which is in charge of computing a hypothesis of diagnosis regarding the inhabitant’s cognitive health (i.e., cognitively healthy, MCI, or PwD).

The aim of that module is to generate a hypothesis of diagnosis based on the long-term predictions of the vision-based trajectory classifier. Of course, in order to produce a reliable hypothesis, the long-term analysis module needs a sufficient quantity of trajectory classifications. Therefore, it is assumed that the module considers the whole data acquired in a given time period; e.g., all the predictions about the trajectories observed in the previous 30 days. The module analyses the whole history *VTC* of vision-based trajectory classifications produced in that period:

$$\begin{aligned} VTC = \{class_1, class_2, ..., class_m\}, \end{aligned}$$

where the value of $class_i$ can be either *‘cognitively healthy’*, *‘MCI’*, or *‘PwD’*. Also, there is a need to compute the number of predictions of each class in *VTC*. Then, the module outputs the hypothesis corresponding to the most frequent class *fc* as follows:

$$\begin{aligned} fc ={arg\,max}_{class\in D} \, \big | \, \{cl \in VTC: cl=class\} \, \big |, \end{aligned}$$

where $D=\{$‘cognitively healthy’, ‘MCI’, ‘PwD’$\}$.

## Experimental Evaluation

In this section, we report our experimental evaluation which was carried out with real-world locomotion data acquired in an instrumented smart-home from a large set of seniors, including cognitively healthy seniors, PwD, and persons with MCI. Therefore, in the following subsections, we explain the used dataset, the experimental setup, and the achieved results. We also experimentally compare our technique with an existing state-of-the-art method.

**Fig. 5:**
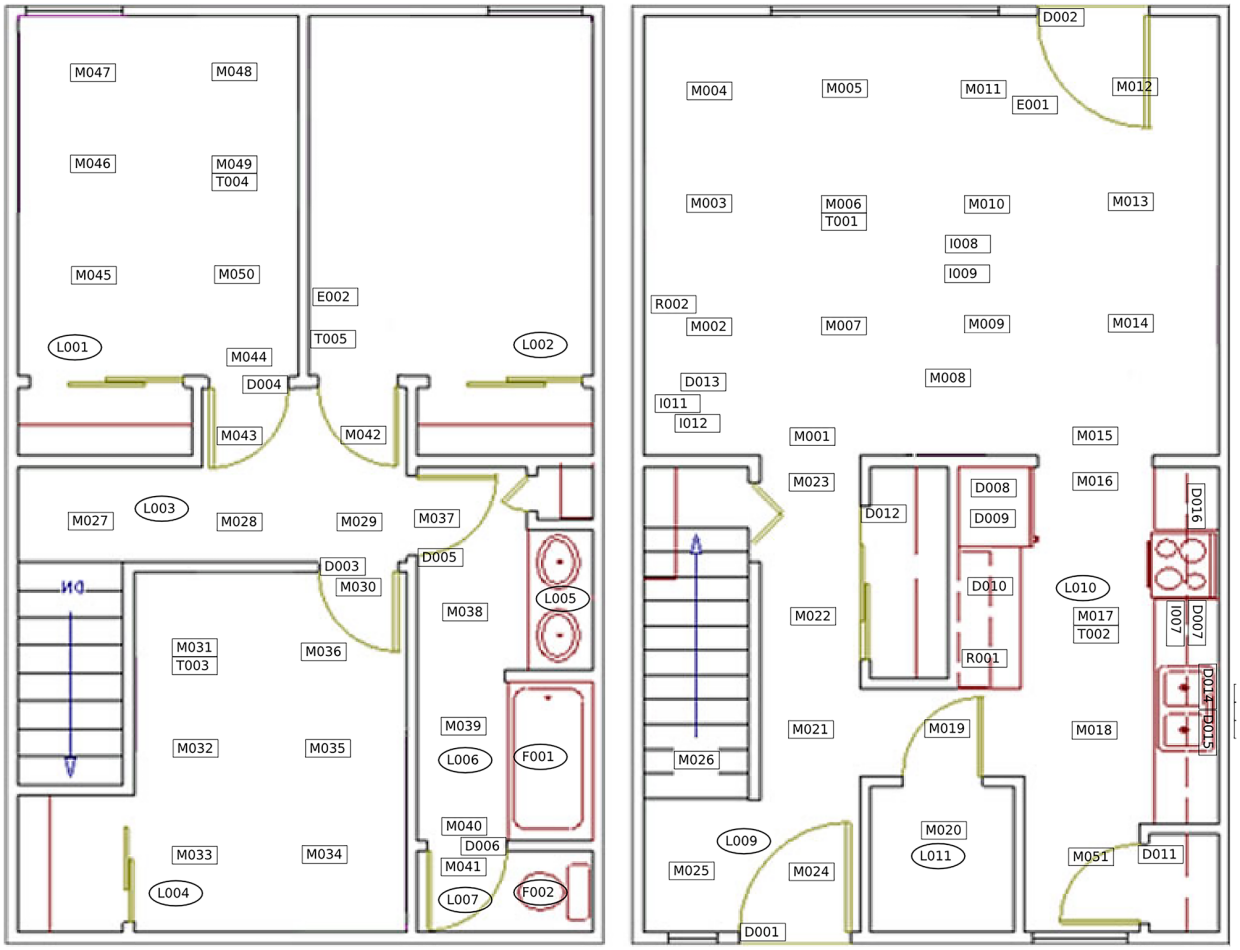
The smart-home layout used in our experiments [43]

### Dataset

The experiments were carried out considering real-world trajectories acquired from 153 individuals in a smart-home of the CASAS test-bed [44] and annotated by the researchers of the Center for Advanced Studies in Adaptive Systems (CASAS) at Washington State University (WSU) [22]. The smart-home layout is represented in Fig. 5. The smart-home is a two-story apartment equipped with different kinds of sensors, and its floor plan includes a living/dining room, three bedrooms, a kitchen, and a bathroom. For our experiments, we relied on passive infrared (PIR) motion sensors and door sensors to track the movements of the individuals in the home. PIR sensors are mounted on the ceiling and their accuracy is about one meter. In total, the apartment included 51 motion sensors and 16 door sensors.

Participants were recruited by advertisement and physician referrals. After obtaining informed consent, participants underwent multidimensional clinical assessment by neuropsychologists, in order to assess the cognitive health status. For the sake of anonymity, explicit identifiers of the individuals involved in the study were removed, and quasi-identifier personal data, such as age, were generalized to age ranges. The protocol of recruiting and data collection was approved by the Institutional Review Board of WSU [22]. As a consequence of clinical examination, each participant was classified as either PwD, person with MCI, or cognitively healthy person.

Participants were asked to individually execute scripted Day-Out Tasks (DOTs) in the smart-home test-bed. DOTs are naturalistic tasks involving the execution of interleaved activities for reaching a certain goal [45]. Each participant executed the DOTs in a single day for an average of three hours. Data collection occurred in the morning or in the early afternoon. During the execution of DOTs, the sensor infrastructure acquired the sensor data triggered by their actions and movements. The detailed descriptions of DOTs, together with the collected dataset, are available on the Web^1^. The smart-home setup is described in detail in [46].

While 40 PwD were recruited, only part of them were able to participate to the data collection in the smart-home. Hence, in our experiments we considered only PwD who were able to carry out activities in the home (19 individuals). In our experiments, we considered also the data acquired from the 80 seniors aged 60 to 74 years old, and from the 54 persons with MCI.

### Comparison with State-of-the-Art Methods

In order to experimentally compare our method with the state of the art, we implemented both a baseline numeric feature extraction method, and the image-based method proposed by Gochoo et al. in [37].

#### State-of-the-Art Numeric Feature Extraction (NFE)

As a comparison, we implemented a baseline feature extraction method, in which each feature corresponds to a locomotion-based clinical indicator of cognitive decline proposed in the literature. We call this method *numeric feature extraction* (NFE). We consider the following indicators:

- *Pacing* [13] travel pattern, defined in the Martino-Saltzman model: this feature counts the number of observations of this pattern in the last day.
- *Lapping* [13] travel pattern, defined in the Martino–Saltzman model: This feature counts the number of observations of this pattern in the last day.
- *Random* [13] travel pattern, defined in the Martino-Saltzman model: This feature counts the number of observations of this pattern in the last day.
- *Jerk* [15] is computed as the first time derivative of acceleration. This features represents the average jerk observed in the individual’s trajectories in the last day.
- *Straightness* [16] represents the average straightness computed on the individual’s trajectories of the last day.
- *Sharp angles* [6] feature counts the number of sharp angles observed in the individual’s trajectories during the last day.

In order to compute the above-mentioned features, we adopt the algorithms recently presented in [47].

#### State-of-the-Art Visual Feature Extraction (GVFE)

In the original paper where the method proposed by Gochoo et al. was presented [37], the GVFE technique was used to recognize those abnormal locomotion patterns that are strong indicators of neurocognitive diseases according to the Martino–Saltzman model; i.e., pacing, lapping, and random patterns. The authors experimented their GVFE technique in the same smart-home environment used in our experiments, with data acquired from a cognitively healthy senior during 21 months. They obtained very good results, achieving accuracy above 97%. Those results indicate that the GVFE technique is effective in recognizing abnormal travel patterns that indicate cognitive impairment. For this reason, we experimentally compare the GVFE technique with our visual feature extraction method.

In the GVFE technique, images in each trajectory are prepared based on the sequence of activation of the position sensors. Each trajectory is converted to a binary image, where the *x* axis represents the temporal order of the sensor activation, and the *y* axis represents the numeric identifier of the fired sensors. For example, suppose that the temporal sequence of activated motion sensors in a trajectory is: M005, M003, M005, M010, M011, M001. Then, the only non-zero pixels in the image correspond to the following coordinates: (1,5), (2,3), (3,5), (4,10), (5,11), (6,1).

**Fig. 6:**
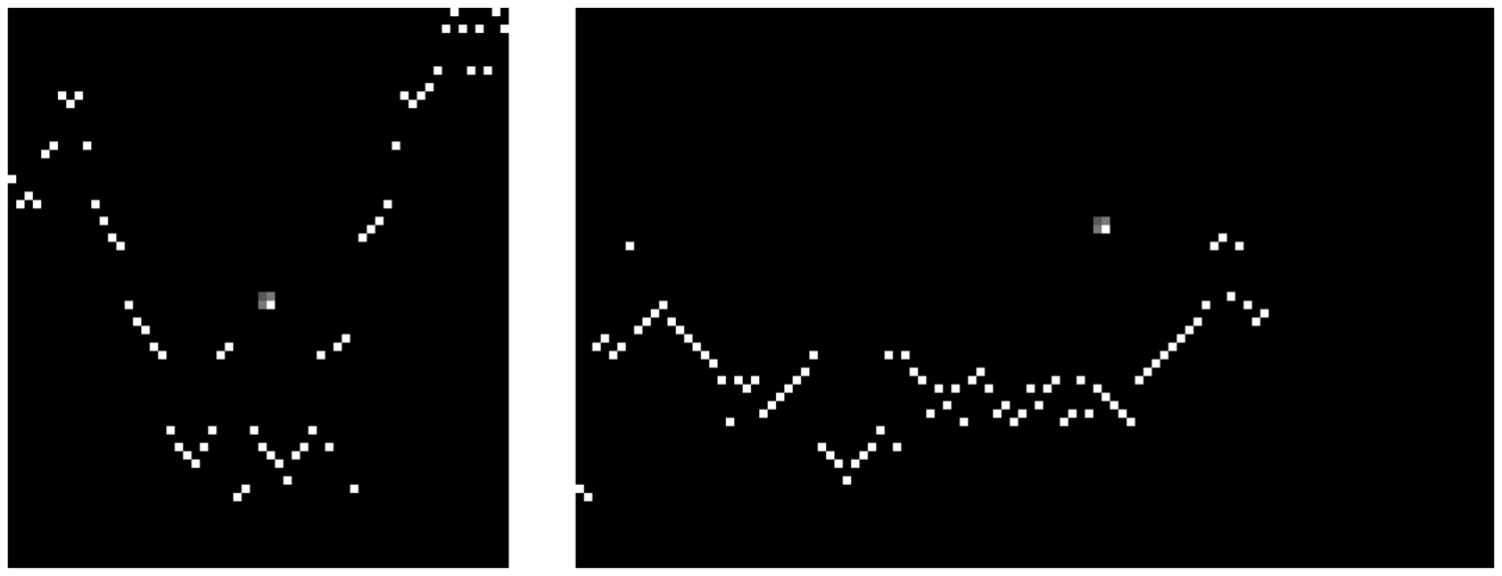
Example of images generated through the GVFE feature extraction method. The left-hand side image is extracted from a trajectory using $T_s = 90 \, s$. The right-hand side image is extracted from a trajectory using $T_s = 150 \, s$

Also for this feature extraction method, we apply trajectory segmentation using different values of the threshold $T_s$. The width of the corresponding images is chosen based on the maximum length of trajectories. For example, in our dataset, using $T_s = 60 \, s$, the maximum length of trajectory for all considered individuals is 32. Hence, images computed using that threshold value have 32 pixels width.

As mentioned before, the *y* axis represents the numerical identifier of the fired sensor. Since in our dataset we consider 67 sensors (i.e., 51 PIR sensors and 16 door sensors), the *y* axis includes 67 different values. Consequently, the images have 67 pixels height, irrespectively from the value of the threshold $T_s$. For thresholds $T_s$ set to $30 \, s$, $60 \, s$, $90 \, s$, $120 \, s$, and $150 \, s$, the image size is $(14 \times 67)$, $(32 \times 67)$, $(60 \times 67)$, $(110 \times 67)$ and $(169 \times 67)$, respectively. Figure 6 shows two samples of images obtained using different threshold values.

#### State-of-the-Art DNN (DCNN)

**Fig. 7:**
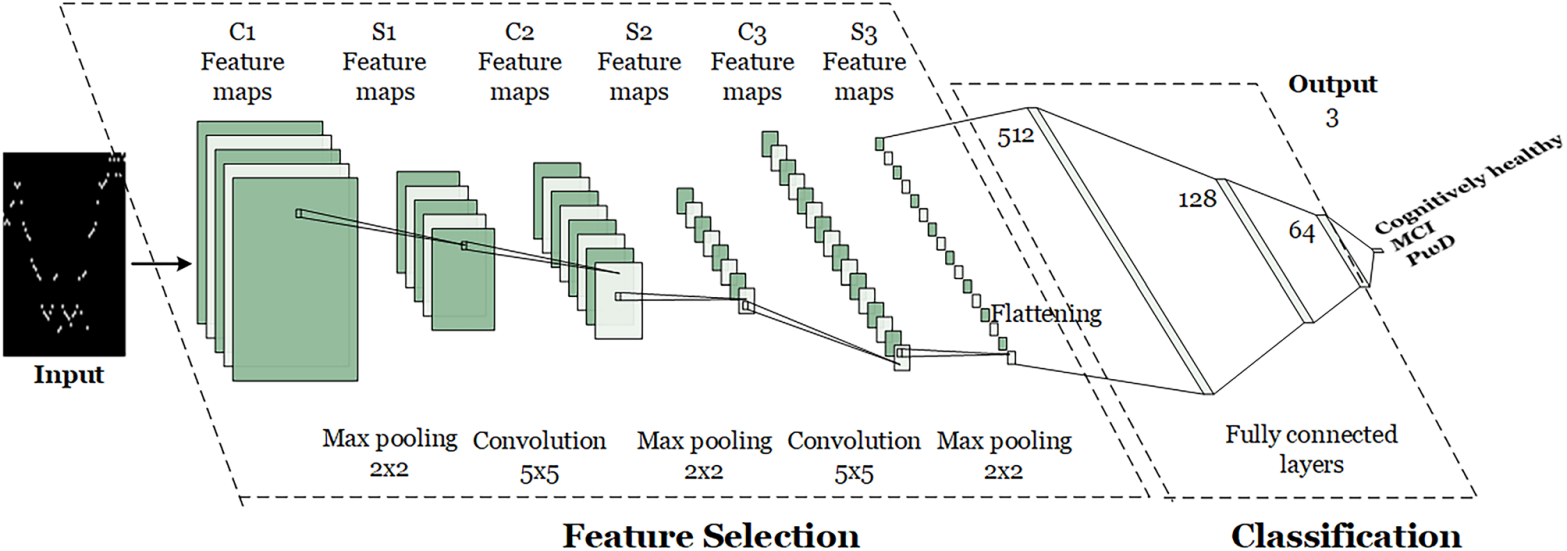
The state-of-the-art deep convolutional neural network (DCNN) used by Gochoo et al. in [37]

In order to compare our DNN architecture with a state-of-the-art one, we consider the Deep Convolutional NN (DCNN) used by Gochoo et al. in [37]. As illustrated in Fig. 7, that network has three zero padding convolution layers and three fully connected layers which are followed by max-pooling layers and feature filters size of $5 \times 5$. The pooling window size is set to 22 and, since max-pooling creates smaller version of input maps, the output images become two times smaller than the input. The first convolution layer has 32 kernels, the second one, which receives the output of the first max-pooling layer as inputs, has 128 feature filters, and the last convolutional layer receives the second max pooling layer output and convolutes them with 256 feature filters.

Finally, in the fully connected part, the layers are flattened, and the output of the third max-pooling layer is converted into a feature vector. In this part, the first, second, and third fully connected layers have 512, 128 and 64 neurons, respectively, and neurons of the last fully connected layer are connected to all three outputs; i.e., cognitively healthy, MCI, PwD. In order to find probability the distribution of the classes, the softmax function is applied. Since the reference paper [37] does not mention internal details such as the used optimizer and its rate, or the chosen loss function, we used the same parameters chosen for our proposed DNN configuration described in Section 5.

### Experimental Setup

We developed all the algorithms in Python. The code is available on the Web^2^. For experimenting the NFE technique, we used the machine learning algorithms implemented by the Weka toolkit [48]. The code for extracting the NFE features is available online^3^. We have used the Python Keras neural network library^4^ to develop the proposed DNN classification systems. In order to support scalability, in the general architecture of our system we envision the use of a cloud-based system for training the DNN model. However, given the relatively small size of the training set used in our experiments, we trained the DNN on a departmental server. We have run experiments on a Linux server with four NVIDIA Tesla p6 graphic boards, a single NVIDIA Pascal GP104 graphics processing unit (GPU), and 16 GB GDDR5 memory. To evaluate the effectiveness of our TRAJ and SPEED visual feature extraction techniques, we have experimented with different values of the $T_s$ threshold for trajectory segmentation, ranging from 30 seconds up to 180 seconds. We also developed the feature extraction method and the DNN used by Gochoo et al. [37] to experimentally compare our methods with the state of the art.

In all the experiments, we applied a *leave one person out* cross-validation approach: we used the data of one individual for the test set, and the data of the other persons for training and validation, iterating on each person to execute the tests on the whole considered participants. With this approach, the data of the same person is never used both for training/validation and test at the same time. For tuning the hyper-parameters of the DNN, training trajectories are split by a fraction of 10% of each category for validation and 90% of them for training.

For the overall performance evaluation, we have used the metrics of macro-precision, macro-recall, and macro-$F_1$ score. These metrics are standard ones for imbalanced problems. In particular, macro-$F_1$ score is a reliable metric in imbalanced cases, since it gives equal weights to the different classes, despite their size. Since the ’accuracy’ performance measure is inappropriate for imbalanced classification problems like the one we are tackling, we do not consider that measure in our evaluation. Indeed, depending on the degree of imbalance, the majority class accuracy value would overcome the accuracy value of the minority classes.

**Fig. 8:**
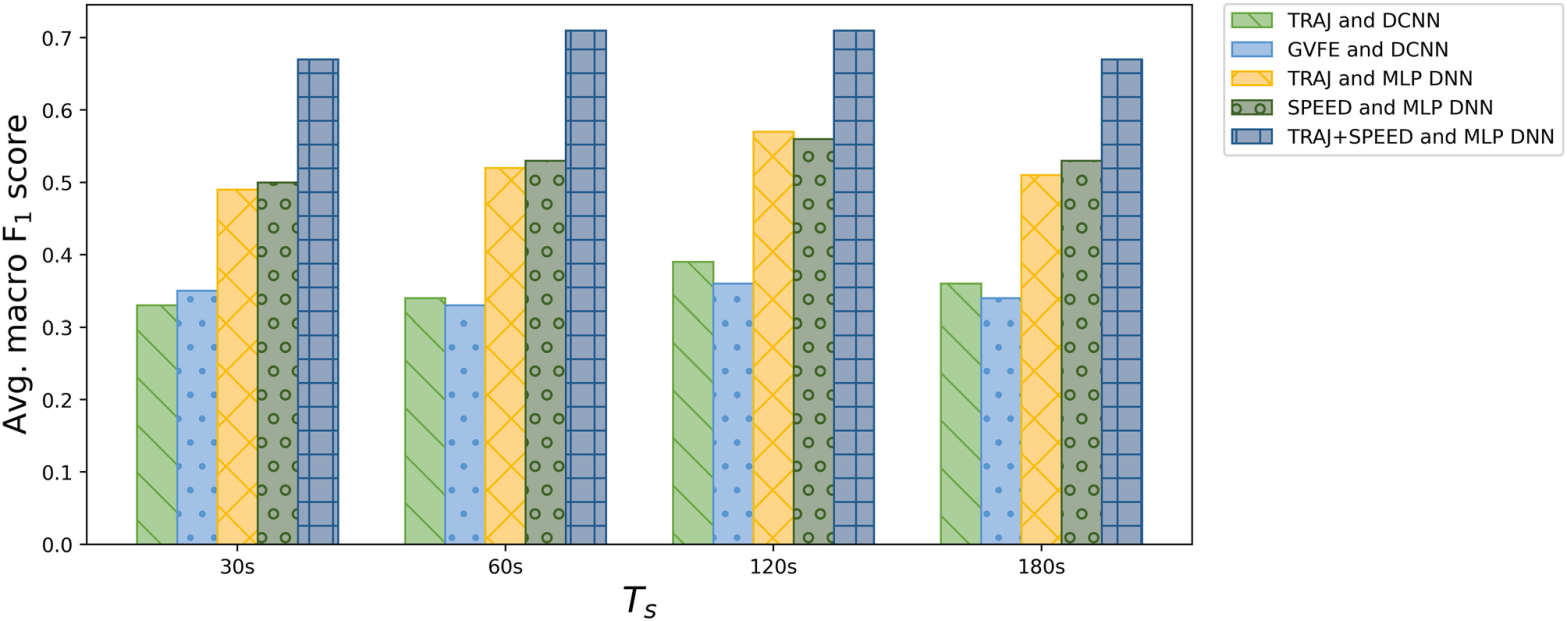
Trajectory images classification: macro-$F_1$ score for the different techniques

### Results of NFE Technique

At first, we evaluated the numeric feature extraction technique explained in Section 6.2.1. For each participant, we built the feature vector using all the trajectory data collected during the day. Each feature vector was labeled with the cognitive status of the individual; i.e., cognitively healthy, MCI, or PwD. We experimented several classifiers:

- The well-known *Naive Bayes* [49] classifier;
- *Logistic regression* classifier [50], relying on a multinomial logistic regression model with a ridge estimator;
- *Multilayer perceptron (MLP)* feed-forward artificial neural network algorithm;
- *Support Vector Machines* [51]
- *k* Nearest Neighbours (*kNN*) [52] lazy classifier, with $k=5$;
- *Ripper* [53] propositional rule learner;
- *C4.5* [54] decision tree;
- *Random tree* [55] classifier.
- *Random forest (RF)* [56] classifier.

Results are reported in Table 1. Overall, the results achieved by the NFE technique are poor. Indeed, all classifiers achieved a macro-average $F_1$ score close to the one of a random classifier. The classifier achieving the best performance in this pool of experiments is the Random forest algorithm, with $F_1$ score of 0.37. These results seem to indicate that, in a home context, numerical statistics about locomotion-based indicators of cognitive decline are ineffective for automatic cognitive assessment. This fact is probably due to the high level of noise introduced by two factors that influence the movement patterns; i.e., obstacles in the home, and execution of daily living activities.

**Table 1:** Results of numeric feature extraction (NFE) method

Class	Measure	Naive Bayes	Logistic regr.	MLP	SVM	
Cognitively	F$_1$ score	0.50	0.69	0.65	0.69	
healthy	Precision	0.56	0.55	0.53	0.53	
	Recall	0.45	0.91	0.84	1.00	
MCI	F$_1$ score	0.46	0.32	0.25	0.04	
	Precision	0.38	0.57	0.39	1.00	
	Recall	0.59	0.22	0.19	0.02	
PwD	F$_1$ score	0.00	n/a	n/a	n/a	
	Precision	0.00	n/a	n/a	n/a	
	Recall	0.00	0.00	0.00	0.00	
Avg.	F$_1$ score	0.32	n/a	n/a	n/a	
	Precision	0.31	n/a	n/a	n/a	
	Recall	0.35	0.38	0.34	0.34	
Class	Measure	kNN	Ripper	C4.5	Rand. tree	RF
Cognitively	F$_1$ score	0.61	0.68	0.59	0.54	0.64
healthy	Precision	0.55	0.53	0.47	0.55	0.56
	Recall	0.68	0.93	0.79	0.54	0.74
MCI	F$_1$ score	0.43	0.21	0.00	0.34	0.39
	Precision	0.46	0.50	0.00	0.35	0.44
	Recall	0.41	0.13	0.00	0.33	0.35
PwD	F$_1$ score	0.00	n/a	0.00	0.00	0.08
	Precision	0.00	n/a	0.00	0.00	0.20
	Recall	0.00	0.00	0.00	0.00	0.05
Avg.	F$_1$ score	0.35	n/a	0.20	0.29	0.37
	Precision	0.34	n/a	0.16	0.30	0.40
	Recall	0.36	0.35	0.26	0.29	0.38

### Results of Single Trajectory Image Classification

At first, we experimentally compare the effectiveness of our TRAJ feature extraction method with the one achieved using the Gochoo’s et al. visual feature extraction method (named GVFE). For this experiment, we used the DCNN used by Gochoo’s et al., named DCNN. In this regard, we want to assess the ability of correctly recognizing the cognitive status of a person by the observation of a single trajectory walked by him/her.

According to the results shown in Table 2, for both techniques, the best results are achieved with the trajectories obtained setting $T_s = 120 s$. As it can be observed in Fig. 8, the technique using TRAJ and DCNN slightly outperforms the one relying on GVFE and DCNN in terms of macro-$F_1$ score.

**Table 2:** Trajectory images classification: results obtained using our TRAJ feature extraction method and DCNN vs GVFE and DCNN

Class	Measure	TRAJ and DCNN				GVFE and DCNN			
		30s	60s	120s	180	30s	60s	120s	180s
Cognitively	F$_1$ score	0.54	0.52	0.57	0.57	0.57	0.57	0.56	0.55
healthy	Precision	0.57	0.53	0.62	0.6	0.62	0.62	0.6	0.57
	Recall	0.51	0.5	0.52	0.53	0.53	0.52	0.53	0.54
MCI	F$_1$ score	0.34	0.36	0.5	0.38	0.36	0.36	0.4	0.35
	Precision	0.33	0.36	0.4	0.37	0.34	0.34	0.39	0.35
	Recall	0.35	0.37	0.41	0.39	0.38	0.38	0.41	0.36
PwD	F$_1$ score	0.12	0.13	0.1	0.12	0.11	0.056	0.13	0.11
	Precision	0.1	0.11	0.08	0.08	0.09	0.04	0.1	0.09
	Recall	0.14	0.15	0.13	0.18	0.15	0.081	0.17	0.13
Avg.	F$_1$ score	0.33	0.34	0.39	0.36	0.35	0.33	0.36	0.34
	Precision	0.33	0.33	0.37	0.35	0.35	0.33	0.36	0.34
	Recall	0.33	0.34	0.35	0.37	0.35	0.33	0.37	0.34

In a second set of experiments, we compare the performance of the TRAJ vs SPEED feature extraction methods, using our MLP DNN. Table 3 shows the achieved results. The two feature extraction methods achieve comparable results. As it can be observed in Fig. 8, for both methods, the best results in terms of average macro-$F_1$ score are obtained using $T_s = 120 s$. It is evident that the results obtained using the TRAJ feature extraction method with our MLP DNN strongly improved with respect to using the DCNN. Indeed, using the MLP DNN we achieve a macro-$F_1$ score larger than 0.57 with less computation time, while the best macro-$F_1$ score obtained using the more complex DCNN was close to 0.36. We believe that this result may depend on the relatively limited size of the training set. Indeed, using a larger training set, the more complex DCNN could possibly outperform the MLP DNN, at the cost of additional time and resource consumption. Due to these results, in the rest of the experiments we use our MLP DNN for performing the classification tasks.

**Table 3:** Trajectory images classification: results of our TRAJ vs SPEED feature extraction methods, using our MLP DNN

Class	Measure	TRAJ and MLP DNN				SPEED and MLP DNN			
		30s	60s	120s	180	30s	60s	120s	180s
Cognitively	F$_1$ score	0.62	0.67	0.67	0.64	0.62	0.66	0.72	0.67
healthy	Precision	0.56	0.65	0.62	0.59	0.56	0.66	0.7	0.64
	Recall	0.68	0.69	0.72	0.69	0.68	0.65	0.74	0.7
MCI	F$_1$ score	0.5	0.55	0.58	0.56	0.53	0.52	0.59	0.57
	Precision	0.46	0.5	0.52	0.52	0.51	0.46	0.55	0.56
	Recall	0.56	0.62	0.65	0.6	0.56	0.61	0.64	0.59
PwD	F$_1$ score	0.35	0.34	0.47	0.33	0.37	0.42	0.39	0.35
	Precision	0.58	0.49	0.75	0.54	0.56	0.56	0.51	0.45
	Recall	0.25	0.27	0.34	0.24	0.28	0.34	0.31	0.28
Avg.	F$_1$ score	0.49	0.52	0.57	0.51	0.50	0.53	0.56	0.53
	Precision	0.53	0.54	0.63	0.55	0.54	0.56	0.58	0.55
	Recall	0.49	0.46	0.57	0.51	0.51	0.53	0.56	0.52

### Results of Two Input Trajectory Images Classification

Since the results obtained using separately the TRAJ and SPEED techniques are encouraging, we perform additional experiments using both TRAJ and SPEED trajectory images as input to our proposed MLP DNN. Indeed, since TRAJ and SPEED images represent different features of trajectories, their combined use may increase recognition rates. This setup corresponds to the TraMiner architecture shown in Figs. 2 and 4.

The achieved results are presented in Table 4. Like in the previous experiments, the best results in terms of macro-$F_1$ score are obtained using $T_s = 120 s$. Also in this experiment, the worse results are obtained using threshold values of 30*s* and 180*s*. Considering these results, as also shown in Fig. 8, it is evident that the combined use of TRAJ and SPEED features significantly improves the recognition performance with respect to the use of the single feature extraction methods. Indeed, the best achieved $F_1$ score is larger than 0.71, while the single feature extraction methods achieve $F_1$ scores close to 0.57.

**Fig. 9:**
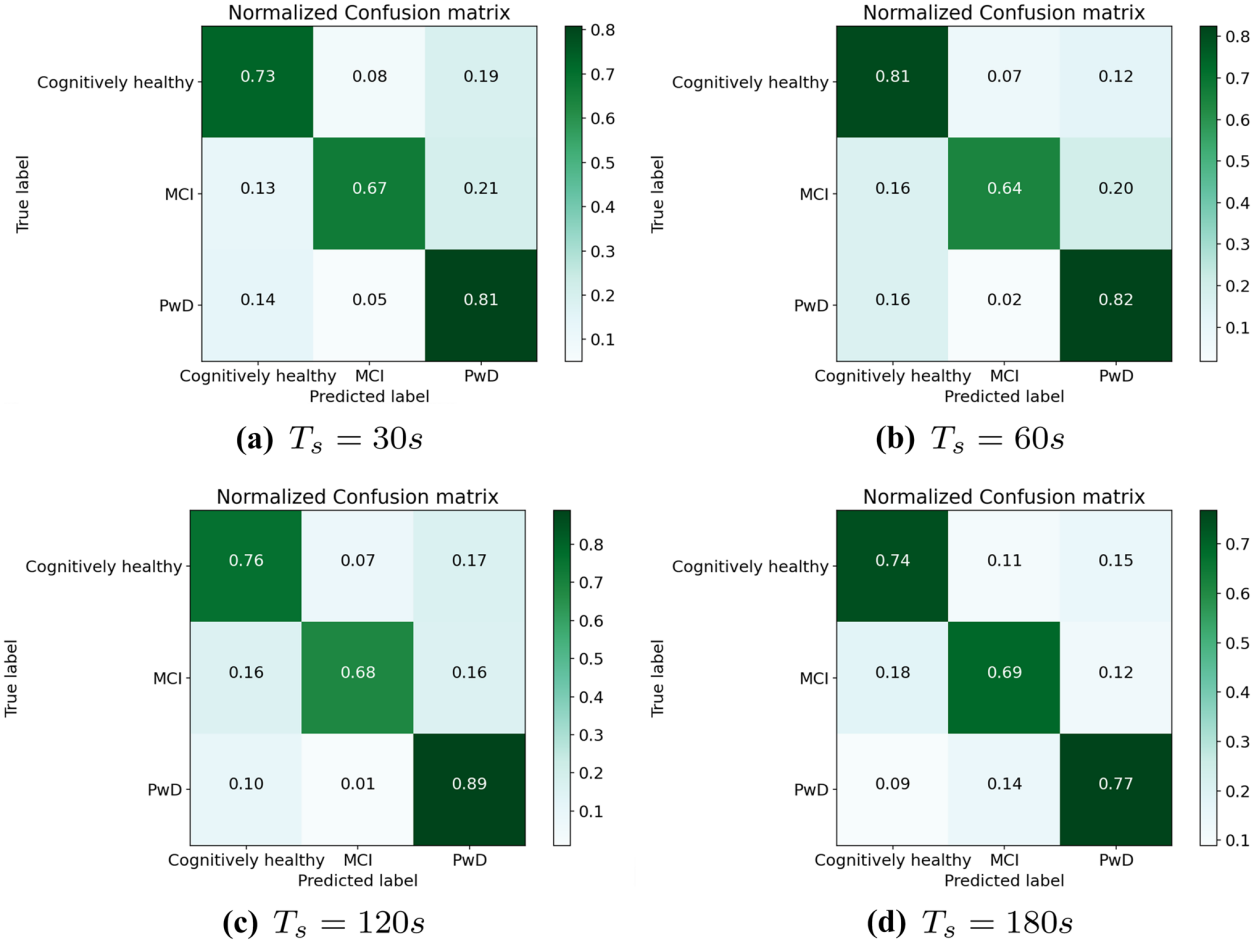
Trajectory images classification: confusion matrices of our TRAJ+SPEED model and MLP DNN

The detailed results can be inspected through the confusion matrices reported in Fig. 9. By observing the confusion matrices, it is evident that the total number of samples changes depending on the chosen value of $T_s$. For instance, with $T_s = 30 s$ we have 5, 195 trajectories, while with $T_s = 180 s$, we have only 540 trajectories. Indeed, in general, the lower the threshold for trajectory segmentation, the larger the number of generated trajectories. Hence, by using larger values of $T_s$ we obtain a smaller number of samples, but we can encode more information in the single trajectory images, since trajectories are generally longer. On the negative side, too large values of $T_s$ may determine very involved images, that may confuse the DNN. On the contrary, too small values of $T_s$ may determine very short trajectories, that do not encode enough information for the DNN. According to our experiments, the value $T_s = 120 s$ provides a good trade-off in this sense.

**Table 4:** Trajectory images classification: results of our TRAJ+SPEED model and MLP DNN

Class	Measure	TRAJ+SPEED and MLP DNN			
		30s	60s	120	180s
Cognitively	F$_1$ score	0.79	0.82	0.8	0.79
healthy	Precision	0.73	0.81	0.76	0.74
	Recall	0.86	0.84	0.85	0.84
MCI	F$_1$ score	0.74	0.74	0.76	0.72
	Precision	0.67	0.64	0.68	0.69
	Recall	0.84	0.86	0.86	0.78
PwD	F$_1$ score	0.49	0.56	0.58	0.52
	Precision	0.81	0.82	0.89	0.39
	Recall	0.35	0.43	0.43	0.77
Avg.	F$_1$ score	0.67	0.71	0.71	0.67
	Precision	0.73	0.75	0.77	0.61
	Recall	0.68	0.71	0.71	0.79

However, the achieved results, which are computed on the classification of single trajectories in isolation, are not sufficient for providing a reliable hypothesis about the cognitive status of the individual. For this reason, in the following experiments we evaluate the performance of the module for long-term trajectory analysis, which considers the whole history of trajectories acquired during a certain period of time.

### Results of Long-Term Trajectory Analysis

**Fig. 10:**
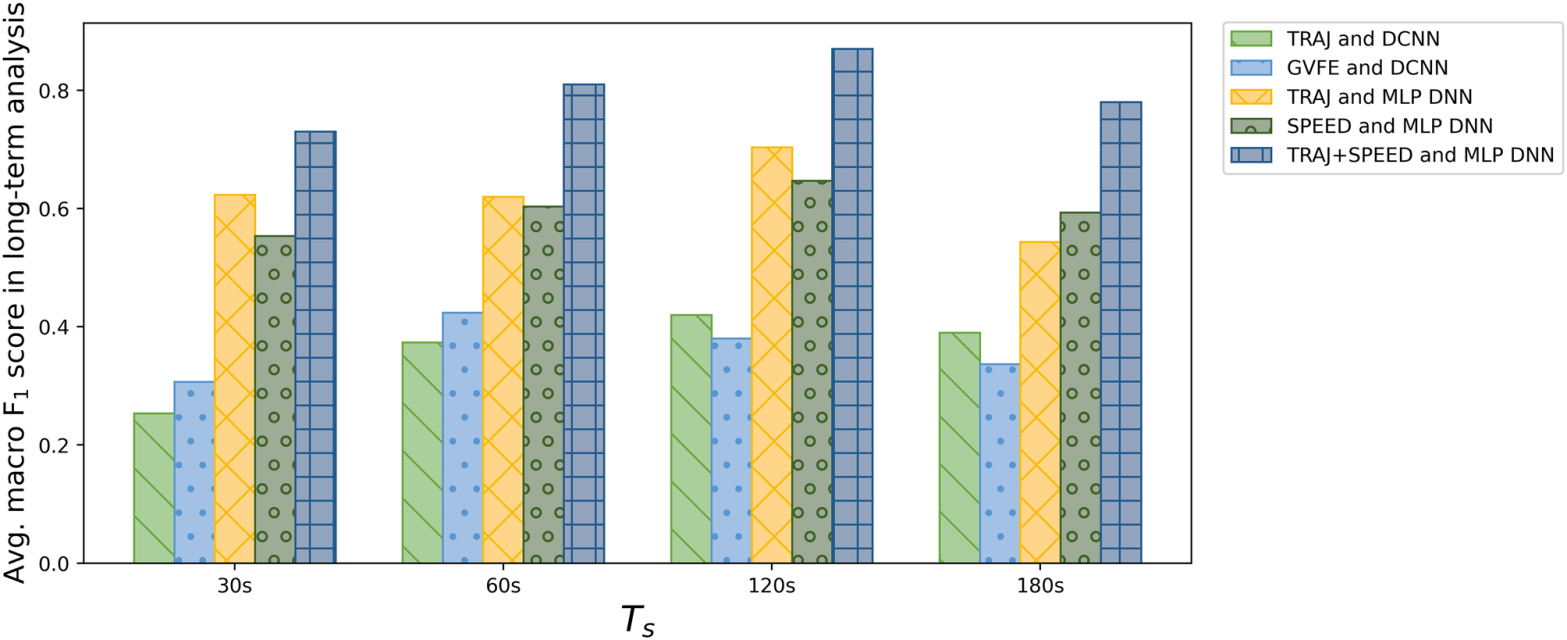
Long-term analysis: macro-$F_1$ score for the different techniques

In these experiments, we apply the algorithm for long-term analysis described in Section 5.2 with all the different techniques for trajectory image classification evaluated in Sections 6.5 and 6.6. Figure 10 provides an overview of the achieved results.

As expected, the results achieved using DCNN with the TRAJ or GVFE feature extraction methods are rather poor. Indeed, those techniques achieve the lowest recognition rates for trajectory image classification. In particular, the TRAJ method with DCNN achieves an $F_1$ score slightly larger than 0.4 with $T_s = 120 s$. Its $F_1$ score is lower with the other values of the threshold. The best result of the GVFE method with DCNN is similar, but obtained with $T_s = 60 s$. Overall, these two techniques do not provide significant results for cognitive assessment, even in the long term.

**Table 5:** Long-term analysis: results obtained using two input trajectory images classification (TRAJ + SPEED features) with our MLP DNN

Class	Measure	TRAJ+SPEED and MLP DNN			
		30s	60s	120	180s
Cognitively	F$_1$ score	0.86	0.93	0.92	0.88
healthy	Precision	0.85	0.94	0.95	0.84
	Recall	0.87	0.93	0.89	0.92
MCI	F$_1$ score	0.78	0.82	0.87	0.87
	Precision	0.69	0.74	0.8	0.83
	Recall	0.9	0.93	0.93	0.9
PwD	F$_1$ score	0.57	0.67	0.83	0.61
	Precision	0.79	0.84	0.89	0.79
	Recall	0.44	0.55	0.77	0.5
Avg.	F$_1$ score	0.73	0.81	0.87	0.78
	Precision	0.77	0.84	0.88	0.82
	Recall	0.73	0.80	0.86	0.77

Results are significantly better using our MLP DNN with the TRAJ or SPEED feature extraction methods. For both techniques, the best results are obtained using $T_s = 120 s$. In particular, the TRAJ method achieves a macro-$F_1$ score of 0.7, while the SPEED method achieves 0.65 macro-$F_1$ score. With these techniques, the increase of recognition performance introduced by the long-term evaluation algorithm is evident. Indeed, both techniques obtain a lower macro-$F_1$ score for trajectory image classification, which is close to 0.57. However, the results obtained with these techniques are still insufficient for providing reliable hypothesis of diagnosis about cognitive assessment.

**Fig. 11:**
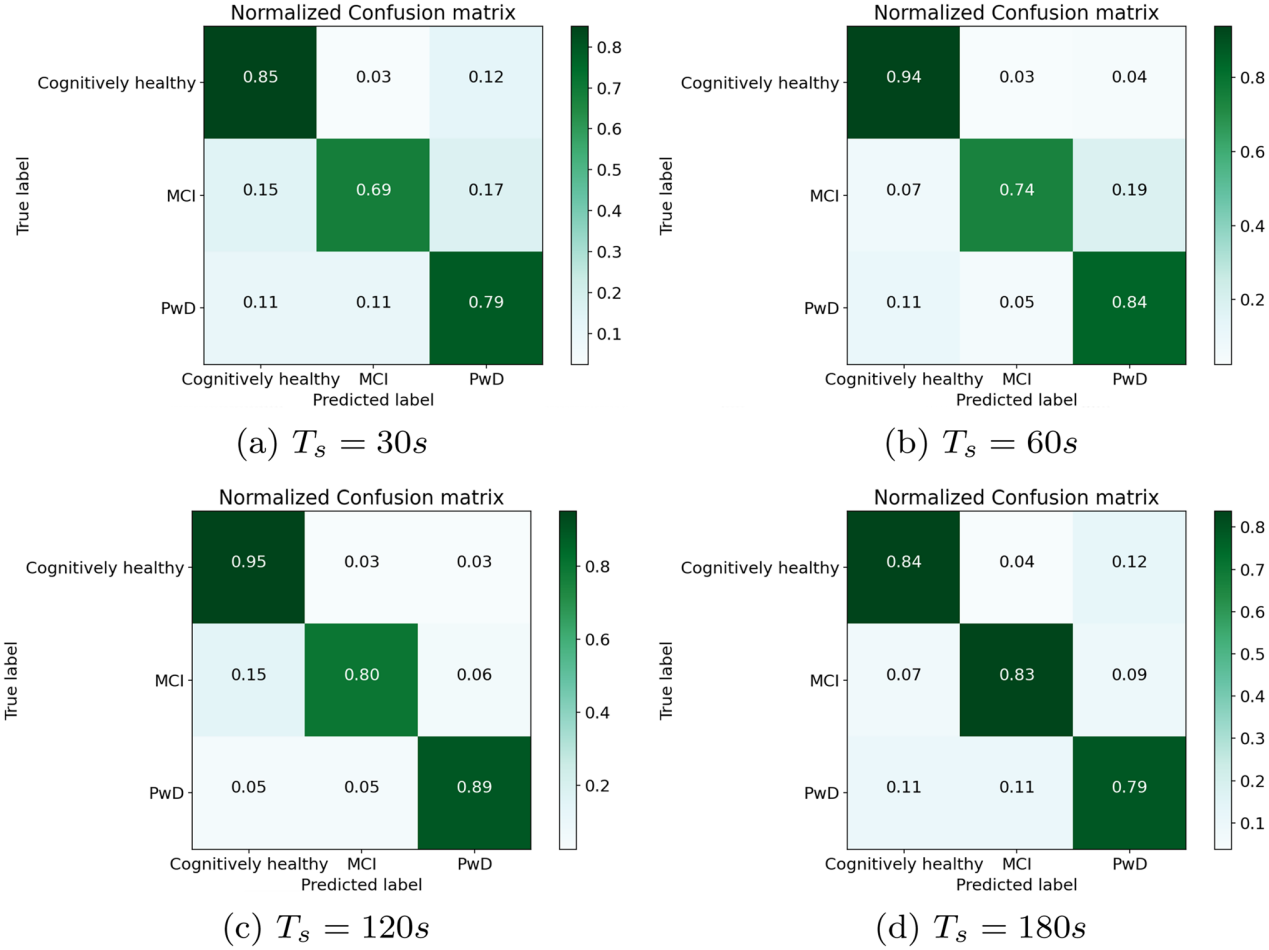
Long-term analysis: confusion matrices obtained using two input trajectory images classification (TRAJ + SPEED features) with our MLP DNN

The best results in this pool of experiments are achieved using two input trajectory images classification (TRAJ + SPEED features) with our MLP DNN. Indeed, the best results are achieved with $T_s = 120 s$, with a macro-$F_1$ score of 0.873. The detailed results are shown in Table 5. As it can be observed, the best results with $T_s = 120 s$ are achieved for the class of cognitively healthy subjects ($F_1$ score = 0.92), which is the most frequent one. The class of MCI subjects obtains $F_1$ score = 0.87, while the class of PwD people achieves $F_1$ score = 0.83.

By closely inspecting the results in the confusion matrices shown in Fig. 11, we can observe that, with $T_s = 120 s$, 17 PwD subjects out of 19 are correctly recognized. Among the other ones, one subject is classified as a person with MCI, and one as a cognitively healthy person. Hence, the false negative rate of PwD is very low. Regarding false-positive predictions of dementia, we observe that three persons with MCI out of 54 are classified as PwD. Out of 80 cognitively healthy subjects, only two are classified as PwD. Hence, the false positive rate of PwD is also low. Regarding the 54 persons with MCI, 43 are correctly recognized, while 8 are classified as cognitively healthy, and 3 of them as PwD. We consider these results positive, since MCI is an intermediate state between cognitive health and dementia, which is difficult to diagnose, especially with automatic tools. Among the 80 cognitively healthy seniors, we achieved only 4 false positives. Indeed, two of them were classified as persons with MCI, and two of them as PwD.

### Dashboard for Clinicians

In order to allow clinicians inspecting the predictions of our system, we have developed a user-friendly dashboard, using the Google Data Studio framework. The dashboard allows inspecting the predictions obtained through the various techniques experimented in our work, achieved using the threshold value $T_s = 120 s$. The dashboard can be freely accessed on the Web^5^.

**Fig. 12:**
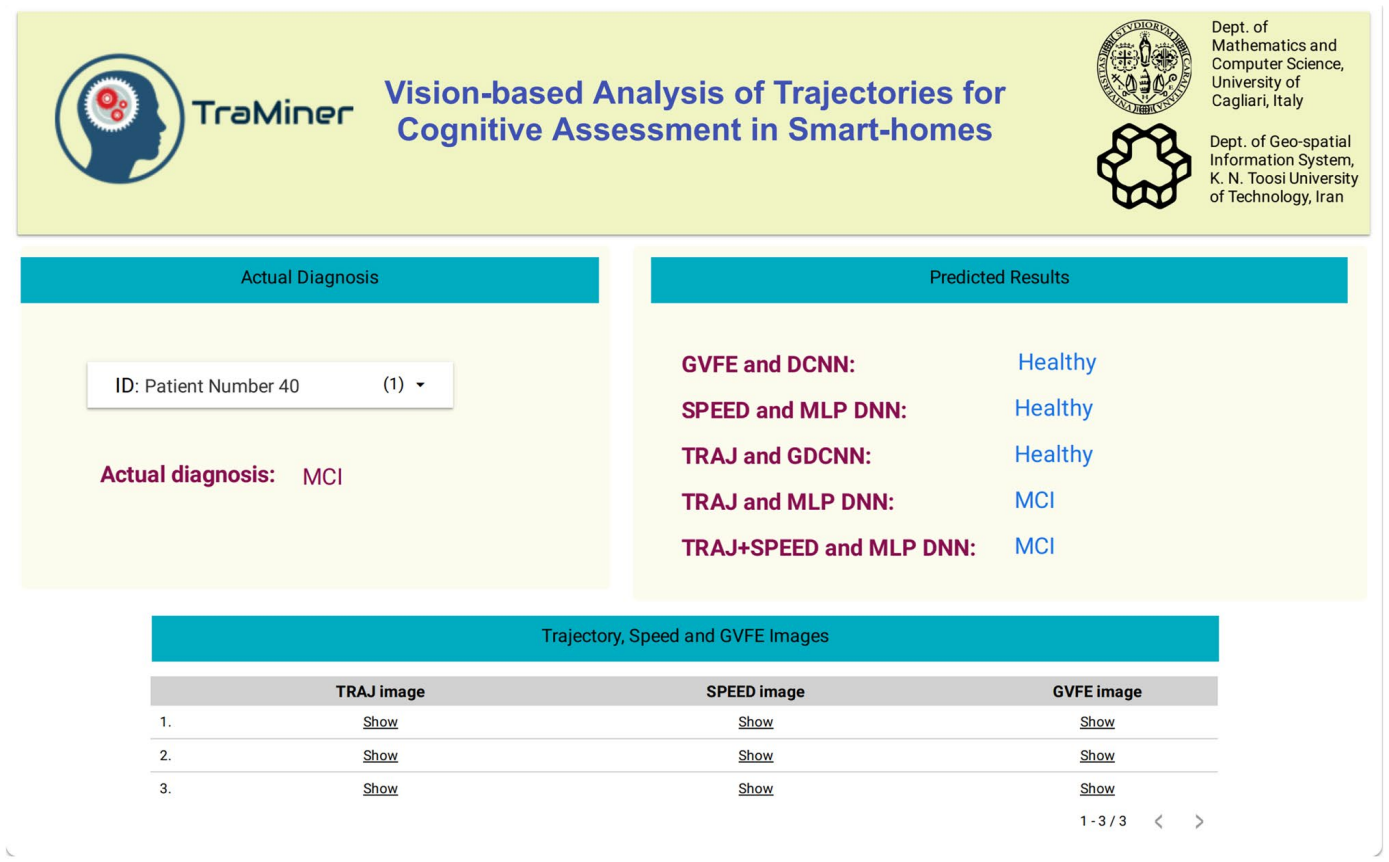
A screenshot of the TraMiner dashboard

A screenshot of the dashboard is illustrated in Fig. 12. The user can select the patient through a drop-down list. The actual diagnosis for the current patient is shown in the left-hand side of the dashboard. On the right-hand side, the user can inspect the predicted diagnosis based on the five different methods: ’GVFE and DCNN’, ’SPEED and MLP DNN’, ’TRAJ and DCNN’, ’TRAJ and MLP DNN’, and ’TRAJ+SPEED and MLP DNN’. We remind that the latter is the actual method implemented by TraMiner, while the other ones are shown only as a reference.

**Fig. 13:**
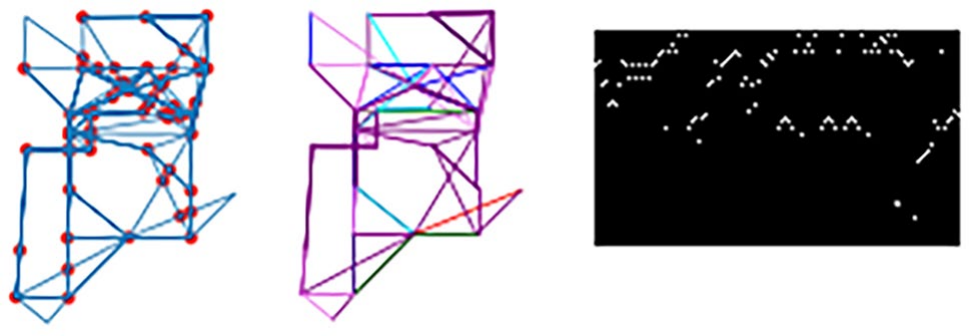
A sample of images for one trajectory in the TraMiner dashboard. From left to right, the images are obtained using the TRAJ, SPEED, and GVFE feature extraction methods, respectively

By selecting a patient, the lower part of the dashboard shows the history of all input trajectories. For each trajectory, the user can visualize the extracted visual features, according to the three experimented methods: ’TRAJ’, ’SPEED’, and ’GVFE’. Those images are available in a table, and can be opened in a separate window through a hyperlink. A sample of three images in the dashboard for one trajectory is shown in Fig. 13.

### Discussion and Limitations

Our experimental evaluation shows that the use of our combined visual features (TRAJ and SPEED), coupled with the use of the MLP DNN, outperforms a state-of-the-art method based on a different visual feature extraction technique and on a more complex DNN. The advantage of our solution consists in the encoding of additional features, such as speed, intersections, and low-level anomaly indicators, that are not captured by existing solutions relying on images. Indeed, those locomotion features are known in the literature to be reliable indicators of cognitive diseases.

In general, our system achieves better results with more frequent classes. Hence, we expect that recognition rates may improve using a training set composed of larger sets of individuals with MCI and PwD. The achieved macro-$F_1$ score suggests that TraMiner may be a useful support for the clinicians to provide a clinical evaluation of the cognitive health status of the elderly. However, this hypothesis should be confirmed by a large trial with the support of clinicians and deployment of our system in real-world conditions.

The experiments show that the recognition performance of TraMiner strongly improves by considering the whole history of trajectories. In this respect, we recall that, despite the dataset having been acquired from more than 150 individuals, each person was monitored only for few hours in one day. Such short observation period may be insufficient to reliably predict the cognitive status of all individuals. Hence, we expect to achieve more accurate predictions by considering a longer history of observations. However, this intuition needs to be verified by additional experiments on a large trial.

A limitation of our vision-based method is the difficulty of manually analyzing the reasons that determined the actual hypothesis of diagnosis by TraMiner. Indeed, it is not straightforward for a human observer to distinguish the trajectory images produced by our feature extraction techniques for the different classes of seniors. In order to provide explainable AI capabilities, TraMiner should be complemented with other methods for cognitive assessment, possibly considering other behavioral models of cognitive decline based on overt [57] or subtle anomalies [58], which are easier to interpret by a domain expert. In order to recognize those behavioral anomalies, TraMiner should be extended with additional sensors and algorithms to recognize activities at a fine-grained level.

Given the nature of the dataset used in our experiments, all the patients’ data were acquired in the same smart-home context. Whether the learned model is portable to a different context is still an open research question. We believe that advanced transfer learning methods specifically designed for image classification may enable training data portability [59], but this aspect should be confirmed by additional experiments with other datasets. Even without the use of transfer learning methods to enhance data portability, our system could be applied to important domains. In particular, a residence for elderly people may consist of several similar apartments. The DNN model could be trained based on the trajectories walked by those inhabitants for which a cognitive status diagnosis is known. That model could be used for the cognitive assessment of the other inhabitants.

## Conclusion and Future Work

In this paper, we tackled the challenging issue of recognizing symptoms of cognitive decline based on the analysis of indoor movements. To this aim, we proposed a technique to extract visual features from indoor trajectories, and a two-input deep learning model for classification. Experiments with a real-world dataset collected from cognitively healthy seniors, people with MCI, and PwD, showed that our system achieves good accuracy for long-term cognitive assessment and outperforms state-of-the-art techniques.

Several directions remain open for future research. Visual feature extraction could be improved by adding some other useful characteristics related to low-level abnormal motion indicators. Our neural network model could be refined to more extensively exploit visual features. The optimal value of the temporal threshold $T_s$ may depend on the kind of activity currently performed by the individual, and by the home shape. Hence, we will investigate techniques to fine-tune $T_s$ considering the current context of the inhabitant. Finally, the system could be provided with explainable AI capabilities by adopting additional models of abnormal behaviors.

## Footnotes

1 http://casas.wsu.edu/datasets/assessmentdata.zip

2 https://sites.unica.it/domusafe/traminer/

3 https://sites.unica.it/domusafe/healthxai/

4 https://keras.io/

5 https://sites.unica.it/domusafe/traminer/
